# Lipoproteins in Cardiovascular Calcification: Potential Targets and Challenges

**DOI:** 10.3389/fcvm.2018.00172

**Published:** 2018-11-23

**Authors:** Yin Tintut, Jeffrey J. Hsu, Linda L. Demer

**Affiliations:** ^1^Department of Medicine, University of California, Los Angeles, Los Angeles, CA, United States; ^2^Department of Physiology, University of California, Los Angeles, Los Angeles, CA, United States; ^3^Department of Orthopaedic Surgery, University of California, Los Angeles, Los Angeles, CA, United States; ^4^Department of Bioengineering, University of California, Los Angeles, Los Angeles, CA, United States

**Keywords:** lipoproteins, calcification, Lp(a), autotaxin, osteogenesis

## Abstract

Previously considered a degenerative process, cardiovascular calcification is now established as an active process that is regulated in several ways by lipids, phospholipids, and lipoproteins. These compounds serve many of the same functions in vascular and valvular calcification as they do in skeletal bone calcification. Hyperlipidemia leads to accumulation of lipoproteins in the subendothelial space of cardiovascular tissues, which leads to formation of mildly oxidized phospholipids, which are known bioactive factors in vascular cell calcification. One lipoprotein of particular interest is Lp(a), which showed genome-wide significance for the presence of aortic valve calcification and stenosis. It carries an important enzyme, autotaxin, which produces lysophosphatidic acid (LPA), and thus has a key role in inflammation among other functions. Matrix vesicles, extruded from the plasma membrane of cells, are the sites of initiation of mineral formation. Phosphatidylserine, a phospholipid in the membranes of matrix vesicles, is believed to complex with calcium and phosphate ions, creating a nidus for hydroxyapatite crystal formation in cardiovascular as well as in skeletal bone mineralization. This review focuses on the contributions of lipids, phospholipids, lipoproteins, and autotaxin in cardiovascular calcification, and discusses possible therapeutic targets.

## Significance of cardiovascular calcification

Previously considered a degenerative process, cardiovascular calcification is now established as a regulated process (1–3) in which vascular and valvular mesenchymal cells undergo osteogenic differentiation (4–8). Clinically, vascular calcification is considered pathognomonic of atherosclerosis. In the coronary arteries, the degree of calcification has been shown to correlate closely with the degree of atherosclerotic plaque burden (9). The presence of calcium deposits in atherosclerotic lesions also appears to increase the risk of intraplaque hemorrhage (10), in which mechanical disruption within a lesion tears microvessels, causing bleeding. The bleeding expands the lesion, so that it may abruptly encroach on the artery lumen causing stenosis or occlusion, which result in ischemia or infarction.

In the aortic valve, calcification carries especially high risk of mortality. Calcific aortic valve disease (CAVD) affects 13% of the population over 65 years of age in the US. Of those who develop symptoms, half die within 2 years (11). Even in the absence of hemodynamically significant obstruction of left ventricular outflow, CAVD is associated with greater risk of cardiovascular events (12). The high morbidity and mortality are due to leaflet stiffening, retraction, and stenosis, which limit valve opening and closing, increasing outflow resistance and oxygen demand, while impairing myocardial perfusion and oxygen supply. Currently, the only options are surgical or trans-catheter interventional replacement of the valve. A wide range of factors and mechanisms are now known to also mediate CAVD, including those in the general categories of hyperlipidemia, inflammation, oxidation, diabetes, apoptosis, hyperphosphatemia, and mechanical forces (13–20). Many of these may act in sequence or in concert, but others are independent (21). This review focuses on the contributions of lipids and the interconnections among lipid metabolism, inflammation, and osteogenesis.

## Biomechanical considerations

Although the mechanisms are not entirely clear, mechanical stresses on the cardiac valves and artery walls, including oscillatory shear on the endothelium and cyclic strain on the valvular interstitial cells and vascular smooth muscle cells, have been implicated in the pathogenesis of cardiovascular calcification (22–24). Cumulatively, these stresses may promote cardiovascular calcification, as they do in skeletal bone, whether through cellular injury leading to dystrophic calcification, or mechanotransduction-related osteogenic differentiation of cells. With regard to lipids, biomechanical stresses may lead to endothelial injury or increased permeability where low shear and oscillatory flows have the capacity to promote accumulation of plasma lipoproteins, such as low-density lipoprotein (LDL) and lipoprotein(a) [Lp(a)]. The severity of calcification in the mitral valve does not correspond with that in the aortic valve, consistent with early work showing that the aortic valve calcifies 10 years earlier than the mitral valve (25). The reason for the mismatch remains unclear, but it suggests a relationship to shear stress or pressure, given that they are exposed to the same oxygen levels and flow volume. Once valve disease begins, the mechanical triggers are likely to worsen. Merryman and Schoen have clarified this reciprocal nature of the interaction between mechanical forces and tissue: while hemodynamic forces affect tissue properties, changes in tissue properties also affect hemodynamic forces (24). Computational models of the valve have been used successfully to predict these interactions (26).

Whether calcium deposits promote plaque rupture or stability is controversial. While plaque rupture into the lumen has been found to occur more often in non-calcified areas of plaque in patients who died from coronary obstruction, the sectioning process itself may disrupt the plaque, especially in areas of lipid pools, where the histologic processing usually removes the lipid. As an alternative, magnetic resonance imaging can detect intraplaque rupture in living patients. Lin et al. performed MRI of carotid arteries in over 100 living patients, and found a strong link (O.R. ≥ 10) between the presence of any type of calcium deposits (whether multiple, surface, or mixed) and intraplaque hemorrhage, after adjusting for age, LDL, maximum wall thickness, and maximum soft plaque thickness, suggesting that calcium mineral deposits promote biomechanical rupture (10). An engineering analysis suggests that the rupture stress is highly concentrated at calcium deposits on the edges that face the direction of stress (27).

In clinical discussions of calcification, the concept of stability is widely used, without careful distinction between its two meanings. Lesion stability may be clinical or biomechanical. Clinical stability refers to the presence and time course of patient signs and symptoms of vascular stenosis or occlusion, such as ischemia, angina, transient neurological symptoms, or infarcts. Biomechanical stability refers to the relative values of tissue mechanical strength vs. tissue mechanical stresses, which is often indirectly inferred from images showing distribution of lesion components, which, as an aside, may improve with machine learning techniques (28). Although these two meanings of stability relate in that biomechanical instability often leads to clinical instability, in investigations pertaining to the effects of treatments on lesions, the two should be clearly distinguished.

## Lipids

The association of serum LDL-cholesterol levels with vascular calcification in patients is well-established (29), and the association is even stronger when the average cholesterol levels are integrated over many years (30). In mice, hyperlipidemia consistently leads to calcification of the aortic root within a few weeks (31). Serum lipidomic analysis in humans has identified fatty acid metabolic markers for cardiovascular calcification. For instance, patients with high coronary calcium scores have more 20:4 fatty acyl chain lipid species and less 18-carbon fatty acyl chain phosphatidylcholines in their serum (32). Bioinformatics approaches have identified phospholipid phosphatase 3 as a key gene in calcific valve disease (33). In vascular cell culture, oxidized lipids induce rapid mineralization (34). As evidence for their importance, use of lipoprotein-deficient serum in the culture medium prevented formation of calcified nodules in the *in vitro* model of vascular cell calcification (35). Neutral lipids accumulate in the sub-endothelial layer of arteries (36). With time, these lipids undergo non-enzymatic modification by the action of products of cellular metabolism (37), such as mildly oxidized phospholipids. These oxidized phospholipids are known to promote calcification *in vitro* through multiple mechanisms. They induce inflammatory cytokines (7), including TNF-alpha, which promotes calcification in part by enhancing BMP-2 activity through inhibition of its inhibitor, Smad6 (7). Modified phospholipids also stimulate calcification by impaired phagocytosis of apoptotic bodies (38). The elastin layer of the artery wall is often the first site of hydroxyapatite crystal formation, especially the ends of fragments, and this may relate to its association with lipids. Elastin is known to act as a sponge for fatty acids (39) and to have high affinity for lipids, LDL, and calcium (40). Thus, pathogenic factors involved in atherogenesis are also involved in calcific disease of the artery wall.

Lipids are also involved in CAVD. As in atherosclerosis, lipid accumulation progresses with age in aortic valves (25, 41). Neutral lipids accumulate in the fibrosa (36). The lesion area, which is usually at the base of the valve leaflet, shows substantial displacement of elastin and thickening of the fibrosa (42). Calcified valves contain sub-endothelial accumulations of lipoproteins ApoB and Apo(a) (36, 42). The earliest calcium deposits are found adjacent to lipoproteins in the deeper regions of the fibrosa along the aortic annulus (42). Lipoprotein deposits are known to undergo non-enzymatic oxidation, and oxidized lipids activate T lymphocytes leading to expression of the oxidized lipid receptor, LOX-1, and proinflammatory cytokines (43, 44). Consistent with this, activated T lymphocytes are also found in the fibrosa layer of the stenotic valves adjacent to calcium deposits (36, 45–47). Even sphingolipid accumulation is associated with calcific valve disease, as seen in patients with Gaucher's sphingolipid storage disease (48). Broadly speaking, it is generally accepted that the mechanisms underlying CAVD are similar to those of atherosclerosis, but they have distinct features, which remain to be clarified. In bioprosthetic valves, lipids also deposit in the spongiosa layer, and the observed lipids (phospholipids, oleic acid, triglycerides, and unesterified cholesterol) are thought to act as nucleation sites for mineral crystals (49).

## Lipoprotein(a) [Lp(a)]

Lp(a) received increased attention in this field in 2013, when Thanassoulis and colleagues reported that a genetic variant in the *LPA* locus encoding Lp(a) showed genome-wide significance for the presence of aortic valve calcification and stenosis across multiple racial and ethnic groups (50). Lp(a) levels have also been associated with cardiovascular risk in a subgroup of patients statins in a clinical trial (51). Lp(a), a lipoprotein particle similar to LDL, is unique in that the apoB-100 protein spanning it has a covalent, disulfide, bond with a glycoprotein, apolipoprotein(a) [apo(a)].

Epidemiologically, Lp(a) levels have a significant positive relation with serum levels of matrix GLA protein (MGP), a known inhibitor of bone morphogenetic protein-2 (BMP-2) (52). If this association were due to Lp(a) induction of MGP, then one would expect Lp(a) to have an inhibitory effect on BMP-2 and an inverse association with vascular calcification. However, Lp(a) is positively associated with coronary calcification, suggesting that the high levels of MGP associated with Lp(a) are due to a negative feedback loop. Patients with the most progression of coronary calcification detected by electron-beam computed tomography (EBCT) imaging also had the highest levels of Lp(a), and patients with the highest levels (>30 mg/dL) of Lp(a) also had the greatest progression of coronary calcification in hypercholesterolemic patients undergoing statin therapy (53).

Calcified human aortic valves have an abundance of Lp(a), and *in vitro* studies have shown that exposure to Lp(a) can promote the chondro-osteogenic phenotype in human aortic valve interstitial cells (54). Interestingly, however, the association of Lp(a) with cardiovascular calcification may be explained by its potential role as a delivery vehicle for pro-calcific and pro-inflammatory factors to sites of endothelial injury. One of its components, apo(a), is a homolog of plasminogen, and as such, can bind to exposed fibrin at areas of denuded or injured endothelium. For unknown reasons, Lp(a) is a preferential carrier of *oxidized* phospholipids (55), and also carries the important enzyme, autotaxin (56), which is discussed in more detail below. Thus, via Lp(a), these compounds may be targeted to sites of endothelial injury, such as occurs on mechanically stressed aortic valve leaflets or atherosclerotic plaque, thereby potentiating the calcification process in these lesions.

## Autotaxin and lysophosphatidic acid

One potential therapeutic target for cardiovascular calcification, lysophosphatidic acid (LPA), is a derivative of oxidized phospholipids, and a potent proinflammatory factor (57, 58). It is produced from lysophosphatidylcholine (LPC) by the action of autotaxin, a lysophospholipase, and another potential therapeutic target. Lp(a), together with autotaxin activity and LPA, are found in human CAVD where they colocalize with oxidized LDL and calcium deposits (56). Autotaxin is also produced and secreted by VICs, and its expression is associated with inflammatory markers in VICs (56). It is widely thought to be involved in calcific atherosclerosis and valvulopathy, but clinical trials have not been completed.

Autotaxin and LPA are also actively considered therapeutic targets in other forms of chronic inflammation, such as idiopathic pulmonary fibrosis and arthritis, as well as in multiple sclerosis and cancer, where it is upregulated (59–61). Genetic deletion of the autotaxin gene is embryonically lethal due to vascular and neuronal defects (62–64). Coincidentally, autotaxin also functions as a phosphodiesterase, ectonucleotide pyrophosphatase/phosphodiesterase-2 (ENPP2) (65). Autotaxin is structurally similar to ectonucleotide pyrophosphatase/phosphodiesterase-1 (ENPP1), a nucleotide pyrophosphatase that generates pyrophosphate, a potent inhibitor of calcification (66). ENPP1 deficiency underlies disorders of ectopic calcification (66, 67). The main structural difference between the two ENPPs is that autotaxin has a lipid-binding pocket and open tunnel, presumably for the fatty acid chain, whereas ENPP1 does not (66). Autotaxin has a rapid turnover (68), and interestingly, in breast tumors, blocking LPA production with a competitive autotaxin inhibitor decreased expression of Nrf2, multidrug-resistant transporters, and antioxidant genes (69), possibly as a feedback response. Autotaxin is known to be bound and inhibited by bile salts (70), raising interesting questions of whether it has any relation to why the famous “Paigen diet” which features the addition of the bile salt, cholate, to a high-fat diet doubles the severity of atherosclerosis (71). From the standpoint of calcification, it is intriguing that, like its relative, ENPP1, autotaxin has the capacity for producing pyrophosphate, an inhibitor of calcification, raising questions about possible pro- and anti-atherogenic effects of autotaxin inhibition.

The role of autotaxin in disease pathogenesis is supported by evidence of chronic inflammation and enhanced rates of breast cancer in autotaxin transgenic mice (72) as well as by the attenuation of rheumatoid arthritis and pulmonary fibrosis in mice with conditional deletion of autotaxin (73, 74). A large number of autotaxin inhibitors have been tested for possible therapeutic use in inflammatory diseases and cancer (75–81). Katsifa et al. have shown that 80% reduction of LPA levels is well-tolerated in autotaxin-null mice or in mice treated with the autotaxin inhibitor, PF8380 (82).

## Statins

Given the substantial links between lipids and cardiovascular calcification, it was anticipated that lipid lowering with statins would be certain to prove to be an effective treatment. Reports from the 1990's and 2000's were supportive of that possibility, although they were less impressive than expected. In mice, lipid lowering by genetic techniques inhibited aortic valve disease (83), but, in humans, the ASTRONOMER trial showed no reduction of aortic stenosis progression by statin therapy in patients with mild to moderate disease (84). Several reasons have been proposed, including the possibility that the disease was already too advanced by the time of treatment, and that a benefit may be seen if treatment were to begin at an earlier stage of disease. It may also indicate that statin treatment has different effects than genetic manipulation. Moreover, statins tend to increase Lp(a) levels (85), which, as noted above, may promote human CAVD. So far, only statins have been tested by a randomized, controlled trial as a possible medical treatment for aortic stenosis.

Compared with mice, larger animal models may provide more accurate measurements of stenosis severity, based on Doppler jet velocity, and may provide physiological conditions more similar to those of humans. For instance, a hamster model of hyperlipidemic–hyperglycemic cardiovascular calcification has been reported (86). In addition, a myocardial infarction-prone strain of Watanabe heritable hyperlipidemic (WHHLMI) rabbits develops more severe aortic valve stenosis (almost 50% reduction) and more evident transvalvular pressure gradients (almost 50% increase) with thickened and degenerated valve leaflets as well as calcified nodules and increased expression of osteogenic factors including Sox9, RANKL, BMP-2, and Runx2 at 30 months of age (86).

In pre-clinical studies, statins were found to reduce progression of calcification in rats with vascular calcification induced by vitamin D and warfarin (87), even though the mechanism of calcification in this pharmacological model is not known to involve lipids. In non-randomized clinical studies, when patients without known coronary artery disease were treated with statins for over a year based on their calcium scores, a decrease in calcification was reported for those who lowered their LDL-cholesterol level below 120 mg/dl (88). In a later study, coronary calcium score progression was significantly reduced, and reversal was seen in patients whose LDL dropped to below 100 mg/dl (89). Later studies found less benefit, including no significant effect of 10 years of lipid-lowering therapy on carotid calcification as measured by MRI (89). Even though prosthetic valve degeneration is independently associated with dyslipidemia in patients (90), and even though direct ethanol-extraction of lipids from bioprosthetic valves reduces their calcification *in vivo* (91–93), statin treatment failed to reduce bioprosthetic valve calcification in patients (94).

In the last decade, compelling evidence is showing the opposite effect, that statins promote vascular calcification. In a London-based clinical study of almost 400 patients with diabetes, Anand and colleagues found that statin treatment was an independent predictor of *progression* of coronary calcification with an OR of 2.3, but not a predictor of acute cardiac events (95). Some suggested that this unexpected result was attributable to higher baseline coronary calcium scores or insufficient lowering of LDL in the statin-treated group (96). However, those potential confounders were excluded in a later VA Diabetes Trial, in which Saremi and colleagues, showed the same results: frequent statin use was associated with accelerated cardiovascular calcification compared with infrequent use, but incidence of cardiovascular events with frequent statin use was not significantly different from those with infrequent statin use over a 4–5 year follow-up (96). Consistent with this, Puri and colleagues used coronary intravascular ultrasound to show that statin treatment promotes coronary atherosclerotic calcification (97). Subsequent reports have consistently tied statin treatment to increased coronary calcification. The Rotterdam study of over 1,700 patients showed statin treatment, at any dosage, was associated with greater calcification, which increased with duration of statin use (98). In the PARADIGM study, statin treatment was associated with more rapid progression of coronary calcification (99). In a study, all 147 patients on statins had progression of coronary calcification (53). Statin-induced calcification occurred even in combination with proprotein convertase subtilisin/kexin type 9 (PCSK9) inhibitors (100). However, patients on a combination of statin and PCSK9-inhibitor therapy had a slower rate of coronary calcification progression compared with statin mono-therapy (100). Mechanisms by which statins may promote coronary calcification remain unclear. Some *in vitro* studies have suggested a direct effect (101), but indirect effects, such as statin-mediated increase in Lp(a) levels (85), are also possible mechanisms.

This has created a dilemma, putting two long established “truths” in conflict: (1) that cardiovascular calcification increases cardiovascular mortality, and (2) that statins reduce cardiovascular mortality. To resolve this, some authors have dismissed or reversed the first. Although the evidence linking vascular calcification and mortality has not changed, some reports assert that the calcification is beneficial, as in “statin use seems to beneficially influence the composition of carotid atherosclerosis” (98) and others have proposed that promotion of calcification is the mechanism for the beneficial effect of statins on mortality (97). Theoretically, the possibility that statins alter biomechanical effects of calcification through changes in specific morphological features cannot be excluded.

This conundrum has translated to clinical care: physicians are advised to tell their patients that coronary calcification is dangerous as the basis for initiating statin therapy, and, later, when the calcification progresses further on the statin therapy, to tell their patients that coronary calcification is protective. Thus, a deeper understanding of the relationships between statin therapy, cardiovascular calcification, and clinical outcomes is necessary.

## Lipids and phospholipids in bone/cartilage calcification

Lipids are also closely associated with bone mineralization. Based on nuclear magnetic resonance (NMR) spectroscopic analysis, vascular calcium deposits, and bone both have fatty-acid lipids entrained in their mineral. These fatty acids may include methyl-branched fatty acids, which would be consistent with lipoprotein particle remnants (102). As noted by Reid et al., “colocalization of mineral and lipid may be coincidental, but it could also reflect an essential mechanistic component of biomineralization” (102). As evidence of their strong association with mineral, phospholipids cannot be totally extracted from calcified tissues, such as bone, until the tissues are decalcified. Among phospholipids, phosphatidylserine is considered the most likely to be involved in initiation of mineralization because of its extremely high binding affinity for Ca^2+^ ions (103). Specific membrane proteins in lipid rafts also participate in mineralization. Phospholipids are even involved in mineralization of dental plaque, the calcium deposits formed on teeth by bacteria (104). The lipid profiles found in human cardiovascular calcification share many features with those in newly mineralized bone and calcified cartilage, particularly the complex acidic phospholipids (105).

## Matrix vesicles

Both bone and vascular mineralization appear to be initiated by matrix vesicles, ~100 nm membrane-bound extracellular vesicles. Phospholipids form the membrane of matrix vesicles (also known as extracellular vesicles), where calcium hydroxyapatite crystal formation is initiated when calcium and phosphate ions interact with phosphatidylserine to form phospholipid-calcium-phosphate complexes. Matrix vesicles are extruded from the outer plasma membrane of cells, which gives them a composition similar to lipid rafts (106), and they are enriched in certain components, including cholesterol, free fatty acids, and sphingomyelin (107). They may also be released by fusion of multivesicular bodies (MVB) with the plasma membrane. Interestingly, lipoproteins may interfere with analyses of matrix vesicle composition, because LDL particles may themselves produce extracellular vesicles (108), and because LDL particles co-pellet with matrix vesicles in these analyses (109, 110).

Matrix vesicles from bone were described poetically by H.C. Anderson as: “protected and controlled internal microenvironments outside cells in which specific metabolic objectives of the host cell may be pursued vigorously at a distance from the host cell” (111). They were originally discovered in 1967 in bone tissue, where they were identified as nucleation sites for hydroxyapatite crystals. Once in the extracellular space, they often bind to collagen, a process that appears to induce alkaline phosphatase activity and calcification (112). Matrix vesicles were found a decade later in calcific atherosclerotic lesions in specimens of human aortas (113). Now they are known to be present in human calcific vasculopathy and valvulopathy (114), and, most likely, they would be found at any sites of physiological or pathophysiological calcification. Matrix vesicles from vascular smooth muscle cells and bone cells both include calcium- and phospholipid-binding proteins, phosphate transporters, and cytoskeletal and surface proteins (115).

Matrix vesicles also mediate a wide range of functions besides initiation of mineralization, such as cell-cell communication, depending on their distinct contents and properties. These may include different microRNA species (miR-122-5p vs. miR-150-5p), different specific phospholipids, different activities of alkaline phosphatase and phospholipase A2, different membrane stiffness, and different membrane receptors (116). Due to these distinct functions and properties, matrix vesicles are now described by a wide variety of names: microvesicles, extracellular membrane vesicles, ectosomes, extracellular vesicles, microparticles, and exosomes, depending on cells of origin, tissue location, and the investigator's field of science.

**Figure 1:**
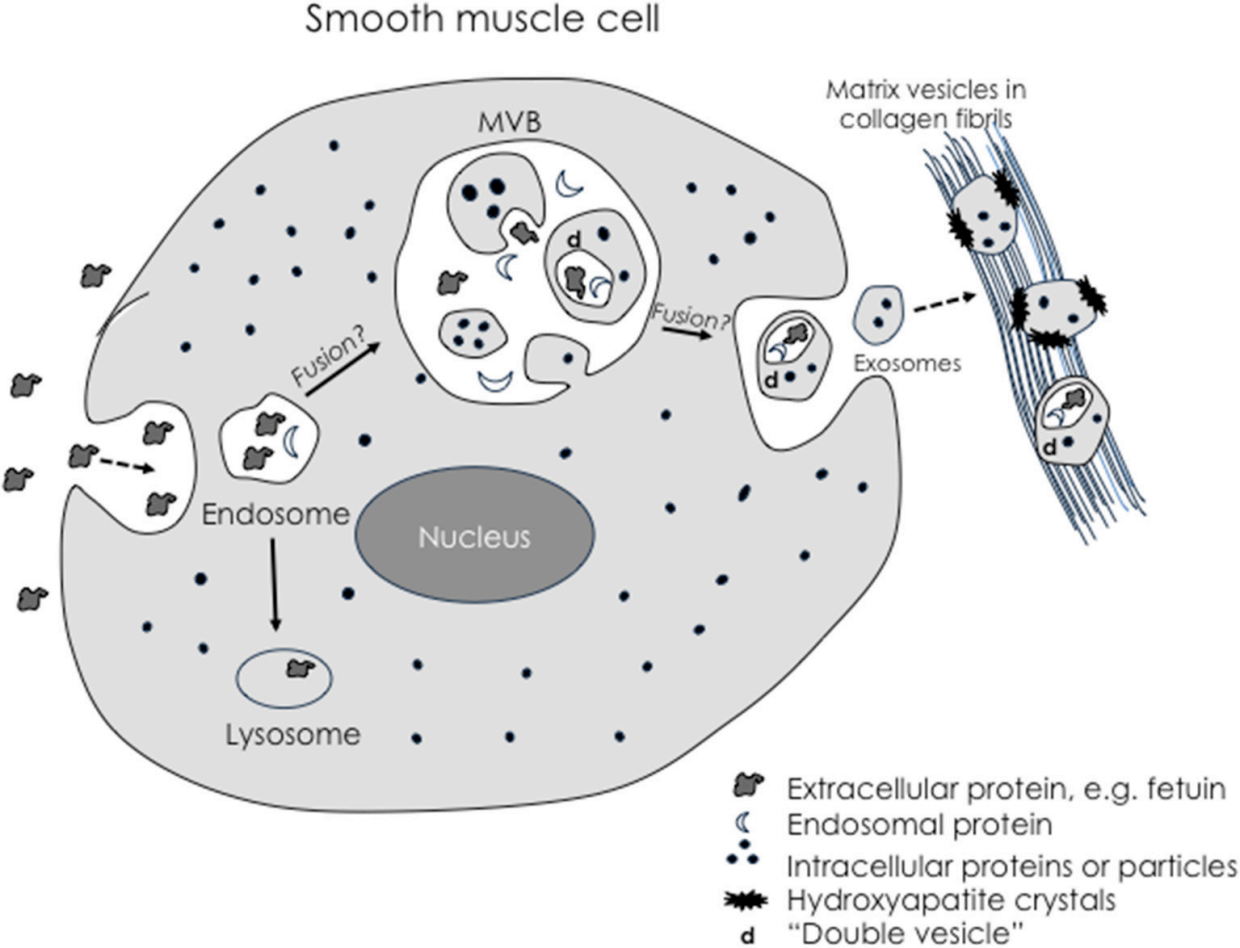
Schematic of possible exosomal biogenesis. Modified from Tintut and Demer (120). Exosomes arise through invagination of the plasma membrane during pinocytosis, which produces endosomes containing extracellular-derived material. Some endosomes may fuse with large multivesicular bodies (MVB). Double membranes are produced when simple microvesicles are formed by a “secondary” evagination of the MVB membrane, which then undergo “tertiary” invagination to engulf extracellular-derived material, creating a double membrane vesicle. When the MVB fuses with the plasma membrane, the microvesicles return to the extracellular space as exosomes. Based on this topological scheme, extracellular particles would be found only in complex exosomes.

A growing consensus is that matrix vesicles are a type of exosome, perhaps the prototypical one, or at least homologous with exosomes (117). Given their tendency to bind to collagen, it has been proposed that they are “anchored exosomes” (118). Exosomes are extracellular, membrane-bound products of the complex endosomal pathway. Their multilamellar membranes (119) may be understood by topological analysis of progressive invagination, fusion, autophagy, and evagination steps of endosomal processing through MVB (Figure 1). Most interesting, given that the multivesicular body may be contiguous at times with the extracellular space, mature matrix vesicles within the multivesicular body may be, nonetheless, topologically exterior to the cell's cytoplasm. As such, they may begin formation of hydroxyapatite crystals while still “deep within” the perimeter of the cell (118). Intracellular vesicles containing calcium phosphate have been demonstrated within osteoblasts—in skeletal bone formation (121), although spontaneous mineral formation within intracellular vesicles may be inhibited where there is an acidic microenvironment.

Of potential clinical significance, the matrix vesicles produced by vascular smooth muscle cells contain alkaline phosphatase that is responsive to active vitamin D (122). The LDL particles that deposit in the subendothelial space carry inactive vitamin D. The vitamin D activating enzyme (1-alpha-hydroxylase) is produced by smooth muscle cells. Thus, LDL accumulation in atherosclerotic plaque may lead to vitamin-D induced induction of alkaline phosphatase, which promotes calcification and raises questions on the potential cardiovascular effects of vitamin D supplementation (123). Vitamin D has even been used to generate rodent models of vascular calcification (124). While these models do produce robust calcific vasculopathy, questions have been raised about whether the mechanisms reflect those of calcific disease found in human atherosclerosis or chronic kidney disease (CKD).

## Potential targets—inhibitors of lipid-related cardiovascular calcification

Inhibitors of oxidant stress, lipid oxidation, and oxidized lipids have been shown to reduce calcific disease. Omega-3 fatty acids reduce osteogenic gene expression in pharmacologically induced medial calcification (125, 126). Activity of a specific enzymatic inhibitor of lipid oxidation, paraoxonase-1, is lower in patients with CAVD and inversely correlated with the severity of the disease (127). A natural antibody to oxidized phospholipids, E06, attenuates both atherosclerosis, and aortic valve calcification in *Ldlr*^−/−^ mice on a high-cholesterol diet (128). Pioglitazone, a ligand for peroxisome proliferator-activated receptor-γ, has been shown to inhibit lipid deposition and calcification in aortic valves of hyperlipidemic mice (129). Interestingly, the effect of pioglitazone was unique to the valve, not affecting the aorta. An inhibitor of oxidant stress, fibulin, also reduces osteogenic differentiation of vascular cells (130). A lipid phosphatase, PTEN has been shown to regulate vascular calcification in multiple models (131), and sodium dichloroacetate, a small molecule inhibitor of AKT, induces vascular cell calcification through activation of p38 MAPK, independently of AKT (132). Small molecule inhibitors of autotaxin are considered potential therapeutic agents in cardiovascular calcification, given that PF8380 [6-(3-(piperazin-1-yl)propanoyl)benzo[d]oxazol-2(3H)-one] (a specific inhibitor of autotaxin) reduces LPA levels in small intestine, liver, and plasma (57, 77).

An important consideration in developing a medical therapy for calcific disease of the cardiovascular system is that treatments that reduce calcium mineral deposits may adversely affect skeletal bone. Epidemiological studies show that calcific vasculopathy and osteoporosis are associated in an age-independent manner (34, 133–139). The simultaneous formation of ectopic mineral suggests that calcium and vitamin D intake may not be the limiting factors in bone formation in osteoporosis. It appears that inflammation promotes calcification in soft tissues, such as in artery, lung, breast, and tendon tissues, but it promotes decalcification in skeletal tissues. An active research question is whether widely used osteoporosis treatments, such as bisphosphonates, which inhibit osteoclastic bone resorption, will affect vascular calcification and in what manner they may do so. The one FDA-approved osteoporosis therapy that promotes anabolic bone formation is teriparatide, an active peptide of parathyroid hormone. Its effect on bone depends dramatically on timing of administration. Repeated intermittent administration is anabolic for bone, whereas sustained high levels are catabolic, as seen in the bone loss in clinical hyperparathyroidism and chronic renal disease. A key question is whether intermittent teriparatide is anabolic or catabolic for the mineral formation in the cardiovascular system. Important mouse studies found prevention of atherosclerotic calcification in hyperlipidemic, hyperglycemic mice (140). Sebastian and colleagues went on to show reduction of medial calcification in the context of CKD, where secondary (2°) hyperparathyroidism was blocked by parathyroidectomy (141). However, treatment of aged hyperlipidemic mice with teriparatide also changes the morphology of pre-existing aortic calcium deposits, raising the possibility that it may affect plaque stability (142).

## Conclusion

Overall, the regulatory mechanisms underlying calcific vasculopathy and valvulopathy are complex, and phospholipids, lipoproteins, and apolipoproteins are prominent among the mediating factors. While medical therapies for cardiovascular calcification have remained elusive, continued study of lipoprotein-mediated pathways hold promise for identifying effective therapeutic targets.

## Author contributions

All authors listed have made a substantial, direct and intellectual contribution to the work, and approved it for publication.

### Conflict of interest statement

The authors declare that the research was conducted in the absence of any commercial or financial relationships that could be construed as a potential conflict of interest.
